# Patients’ and family members’ perspectives on arrhythmias and sudden death in dialysis: the HeartLink focus groups pilot study

**DOI:** 10.1186/s12882-021-02403-0

**Published:** 2021-05-27

**Authors:** Eric J. Xu, LaPricia Lewis Boyer, Bernard G. Jaar, Patti L. Ephraim, Luis Gimenez, Alan Cheng, Jonathan Chrispin, Matthew R. Weir, Dominic Raj, Eliseo Guallar, Tariq Shafi

**Affiliations:** 1 Beth Israel Deaconess Medical Center, MA Boston, USA; 2 Johns Hopkins University School of Medicine, MD Baltimore, USA; 3 University of Maryland, MD Baltimore, USA; 4 George Washington University, Washington, DC, USA; 5 University of Mississippi Medical Center, 2500 North State Street, MS 39216 Jackson, USA

**Keywords:** Patient perspectives, Family perspectives, Focus groups, Dialysis, Arrhythmia, Sudden cardiac death

## Abstract

**Background:**

Patients receiving dialysis face a high risk of cardiovascular disease, arrhythmia and sudden cardiac death. Few patients, however, are aware of this risk. Implantable cardiac monitors are currently available for clinical use and can continuously monitor cardiac rhythms without the need for transvenous leads. Our goal was to gauge patients’ and family members’ perceptions of these risks and to identify their concerns about cardiac monitors.

**Methods:**

Two 90-minute focus groups were conducted: one with patients receiving in-center hemodialysis and one with their family members. Trained moderators assessed: (1) knowledge of cardiovascular disease; (2) cardiovascular disease risk in dialysis; (3) risk of death due to cardiovascular disease; (4) best ways to convey this risk to patients/families; and (5) concerns about cardiac monitors. The sessions were audiotaped, transcribed, and independently analyzed by two reviewers to identify core themes. Emblematic quotations were chosen to illustrate the final themes.

**Results:**

Nine adult patients and three family members participated. Patients felt education was inadequate and had little knowledge of arrhythmias. Patients’/families’ concerns regarding cardiac monitors were related to adverse effects, the notification process, and cosmetic effects. Patients/families felt that nephrologists, not dialysis staff, would be the best source for education.

**Conclusions:**

The preliminary data from this small study population suggest that patients/families are not well aware of the high risk of arrhythmia and sudden cardiac death in dialysis. Further investigation is required to gauge this awareness among patients/families and to assess their impressions of implantable cardiac monitors for arrhythmia detection and management.

**Supplementary Information:**

The online version contains supplementary material available at 10.1186/s12882-021-02403-0.

## Background

Patients initiating dialysis for end-stage kidney disease (ESKD) face a high risk of cardiovascular disease (CVD), arrhythmia, and sudden cardiac death (SCD).[1] Arrhythmia detection by miniaturized implantable cardiac monitors (ICMs)[2–7] may help individualize care by promoting the use of established management strategies for arrhythmias.[8] Few nephrologists and patients, however, are aware of these devices. We report the results of a qualitative study assessing patients’ and family members’ knowledge gaps.

## Methods

As part of a larger study, we developed educational materials to increase ESKD patients’ and family members’ awareness of CVD, arrhythmias, and ICMs (see Supplemental Materials). Participants were recruited from two hemodialysis clinics in Baltimore, Maryland. All participants were age ≥ 18 years, spoke English, and provided oral consent. The project was reviewed by the Johns Hopkins Medicine Institutional Review Board (IRB00133847) and designated as a quality improvement project which allowed waiver of written consent. Two moderators led the focus groups and asked participants open-ended questions. Discussions were audiotaped and transcribed for template analysis.[9].

## Results and discussion

Nine patients (7 male, 2 female; ages 28–67) and 3 family members participated. All patients self-identified as non-Hispanic (8 African -American, 1 White). Patients’ highest completed level of education included high school (3), two years college (2), four years college (1), and graduate/professional school (3). Six themes emerged from the focus groups: knowledge about CVD, knowledge about arrhythmias, family member involvement, source of education about CVD/arrhythmia, impressions about ICMs, and impressions about ICM educational materials.

Overall, we found that patients/family members were aware that there are different types of heart disease, such as hypertension, but had much less knowledge about arrhythmia. As demonstrated here, patients experience arrhythmia differently and at times do not realize that their symptoms reflect complex pathophysiology involving the kidneys, heart, and dialysis. Arrhythmia and sudden cardiac death are the leading causes of mortality for ESKD patients receiving dialysis and our results imply that patients and their families do not fully understand these risks (Table 1). Notably, appreciation of these risks often occurred after being notified by a physician in an urgent/emergent setting or after developing sequelae of untreated CVD. Additionally, many arrhythmic episodes are asymptomatic. Thus, in addition to educating patients about common clinical presentations, arrhythmia management in the dialysis population may also be improved by cardiac monitoring programs that allow for the early detection of otherwise “silent” pathologic rhythms.

**Table 1:** Patient and family member quotes

Theme	ESKD Patient Quotes	Family Member Quotes
**Knowledge about CVD**	“…they don’t actually tell you exactly what dialysis is and how it really affects your heart.” “When they first tell me I have to go to the heart doctor, I automatically was, like, what? What happened?” “Blood pressure also brought me in…because that took me to the stroke…I had a stroke, and I stood there for three days…I didn’t realize it. I had lost three days, and my pressure was 290 over 190.” “I was 280 and I went to the doctors and he said, you got diabetes, high blood pressure and it was high cholesterol…I made a joke out of it…I still do whatever I want to do…They say, don’t eat this, don’t eat that. The same exact thing I did before I got on dialysis, I do right now…I drink a six pack of coca cola a day…I don’t feel no different. When my doctor tell me I had to go on dialysis and they was telling me all the symptoms, I never had them symptoms.”	“For a dialysis patient, to be honest, all you think about is the kidney. That’s it. You don’t think about the heart, but then I started to hear on TV a lot of people died of heart attack while on the machines, a lot of dialysis patients.” “[He] has been on dialysis for a while and…just had a big scare two weeks ago…He said, Ma, I don’t know what it was, but I felt like I was pressing myself to walk, pressing myself to walk…it happened again, and when he came to dialysis, the doctor came to him and said [your] potassium level was dangerously high…your heart could have stopped.
**Knowledge about arrhythmias**	“I never felt it happen…my heart would go from like 80 beats a minute up to over 130 for about 15 seconds and drop back down. That’s the arrhythmia that they discovered, but I didn’t even know I had it.” “I have to tell you how I discovered that I had this arrhythmia… My heart rate was 138…then the doctor came in and said, you got this arrhythmia going on.” “So I don’t have heart disease, but I have A-Fib…I had two strokes, so I…have a…10 percent chance of having another one.” “Is arrhythmia and A-Fib two different things?”	“No.” (referring to awareness of arrhythmias)
**Family member and caregiver involvement**	“…my girl and my sister, basically they take care of all that…when my doctor talking, my girl ask questions and all that.” “I was saying that they need a pamphlet. Yeah, give them one also.”	“Every step of the way. I want to be there at the consultation. I want to be there…like his backup.” “I would say…dialysis patients tend to hide a lot of things that they don’t want you to know…[he] never discussed any of this with us until he got that scare.”
**Preferences regarding source of education**	“I don’t think the dialysis staff would know.” “Half of [the dialysis staff] don’t know.” “Of course we’re going to have questions, and if they can’t answer the questions, what are we doing?” “…only the doctor can answer some questions.”	“I think the doctor should be the one…to bring it up.”
**Impressions about ICMs**	“what if it were to malfunction? What would it do to me?” “What is the side effects, actually?” “…when I look at myself, would I be able to see a big knot there?” “…would it leave a scar? NO more battle scars.” “How long would the procedure take?” “I’m sure [the monitor] might complicate the catheter.” “I also think that there are a lot of people who are on dialysis who have major, major heart problems who would have a problem with this procedure, even though it only takes a minute…that’s the reason I couldn’t have some stuff done on me is because of my heart problems. The doctor wouldn’t take the chance.” “It’s a tracking device. It’s for a government, if you don’t pay your bills, they shock your heart. They testing it on us, first.” “If you’re going through a medical detector, would that alarm there, or if you’re on a plane, would the air pressure affect it there?” “What about swimming and showering?” “Suppose, [the] cardiologist starts to get bad signs from this thing…Would he want to call me in…and discuss what’s going on here?” “Will it save your life? Let’s say I’m getting ready to have a heart attack in 15 minutes…”	“…how long it going to take for them to heal from the procedure?” “Infection.” “Can he go through the airport with it?” “I know the doctors have the information, but is there going to be like a website for the patients to go and track their own information?”
**Impressions about educational materials**	“If I was interested in participating, this would be helpful. There is a number, there’s who is conducting the survey, what the survey is pertaining to, how long, everything. It’s pretty, it scratches the surface, which is what I would need to want to dial this number.” “It’s clear…it’s not too medical term. It’s easy to for anybody. A child could read this.” “It’s missing one thing for me? Pros and cons.” “It should say have your questions ready, you know?” “I think one of the things I would put, the main thing, speak to your cardiologist or nephrologist and understand how and why this might be something for you.”	“I think if you put it up in dialysis sites and stuff like that, it would catch my eye right off.” “It gets straight to the point.” “It’s not something that we would just brush past.”

Our focus group suggested that patients and family members were overall intrigued by ICMs and found the educational materials straightforward. The majority of concerns were related to adverse and cosmetic effects, as well as the event notification process itself. Comments from participants suggested that ICM enrollment could be improved by addressing such concerns in educational materials; for example, by including a “pros and cons” section as well as a reminder to speak to providers about device eligibility (Table 1). In addition, patients and family members expressed uncertainty over whether dialysis staff would be able to answer questions regarding arrhythmia/SCD and indicated that they would prefer receiving this education from physicians. These results underscore the role of the nephrologist in patient education. While the management of “traditional” complications of ESKD (e.g. hypertension, anemia, hyperphosphatemia) is standardized within Nephrology, management strategies for associated cardiac events such as arrhythmia/SCD are not well defined. Efforts to improve ICM adoption among dialysis patients could be aided by a “Cardiorenal” curriculum that includes procedural skills for monitor implantation and arrhythmia identification and management.[8] Implementing such a curriculum for trainees could help prepare future nephrologists in addressing patients’ concerns and knowledge gaps about arrhythmia/SCD.

In our study, patients and their family members agreed that families should be present during dialysis education. Notably, family members observed that dialysis patients sometimes withhold disclosing new symptoms, suggesting perhaps that while patients generally do want families to be involved in their care, there is gradation to the extent that this involvement is desired by patients (Table 1). Such behavior, though not explored in this study, can possibly be better understood when considering the psychological burden of dialysis. As documented previously in the literature and noted in our clinical experience, dialysis initiation is an emotionally traumatic event that carries the stigma of illness.[10] Although beyond the scope of this study, further work investigating the psychosocial challenges faced by dialysis patients could help clarify how certain patients, rather than being “non-compliant”, are simply overburdened by disease. Our findings highlight the important task nephrologists undertake in not only educating patients and their families, but also in helping them navigate the intangible psychosocial complexities of ESKD.

Our study contains limitations worth mentioning. First, the number of participants was small, likely limiting the range of discussion topics. A larger study with more patients and family members could possibly clarify beliefs and behaviors that our study did not address; for example, why a patient/family member did not think that hypertension or palpitations are related to dialysis, or why a patient chose not to tell their family about possible symptoms. Second, patients were recruited from two urban dialysis units; the medical literacy, experiences, and concerns of the participants might not match those of patients/families from other geographic areas. Third, not all patients attended the focus group with family. We did not collect information on the reasons for non-attendance, which could include a combination of competing time commitments, caregiver burden, or lack of participation in patients’ healthcare decisions. Given the unique perspective provided by family members, their absence limits the generalizability of our study. Although 3 family member participants is a small number, we believe that a small focus group around a highly specialized area of knowledge and experience is preferable over a survey design.[11] Future focus groups can build on our preliminary data by recruiting more participants to allow for a more comprehensive exploration of patient and family perceptions.

## Conclusions

To our knowledge, this is the first study to explore perceptions of arrhythmia and SCD risk among dialysis patients and their families (Table 2). Our results suggest that patients lack knowledge around these topics and illustrates the role of nephrologists to address this gap. Nonetheless, given the small-scale nature of our study, our work is preliminary and further investigation is needed to corroborate our findings.

**Table 2:** Findings and Implications

Findings	Implications
Patients and their families were minimally aware of the risk of CVD, arrhythmia, and SCD in dialysis and had limited knowledge about the variable presentation of arrhythmic episodes.	Patient education in nephrology practice and dialysis facilities needs to emphasize the risk of arrhythmia/SCD and its common presenting symptoms.
Both patients and families see nephrologists as the primary educators and coordinators of ESKD care. However, there is residual confusion regarding the overlapping roles of Nephrology and Cardiology in managing CVD in the setting of ESKD.	Nephrologists need to clarify their role in the management of arrhythmias occurring in the setting of dialysis treatments.
Arrhythmic episodes are often asymptomatic and patients tend to underreport symptoms.	Efforts to improve long-term survival on dialysis cannot be achieved solely by increasing arrhythmia/SCD awareness among patients without a concurrent strategy for arrhythmia detection
Implantable cardiac monitors concerns primarily limited to adverse effects, the notification process, and cosmetic consequences.	Patients and their families were intrigued by ICMs and would be willing to undergo implantation if the physical harms were outweighed by the benefits of early detection.
Patients asked whether ICMs could deliver therapy.	Patients desire active management of arrhythmias when detected.
Patients and families were unaware of the existence and capabilities of ICMs.	Nephrologists and dialysis facility staff may not be aware of current technologies for arrhythmia monitoring. Widespread clinical use of ICMs will require additional efforts to educate patients and providers.

## Supplementary Information

**Additional file 1.**

## Data Availability

Data sharing is not applicable to this article as no datasets were generated or analyzed during the current study.

## Abbreviations

ESKD: End-Stage Kidney Disease.
CVD: Cardiovascular Disease.
SCD: Sudden Cardiac Death.
ICM: Implantable Cardiac Monitor.
