# Supplementary material for: Patients’ and family members’ perspectives on arrhythmias and sudden death in dialysis: the HeartLink focus groups pilot study
Source: BMC Nephrol. 2021 May 27;22:199. doi: 10.1186/s12882-021-02403-0 (PMC8161918; doi:10.1186/s12882-021-02403-0)
Supplement: Supplementary file 1 — Additional file 1. [file 12882_2021_2403_MOESM1_ESM.docx]

**Supplementary Materials: Methods**

*Overall study design*

As part of a larger study designed to understand the burden and consequences of arrhythmias in dialysis patients (NHLBI: R01HL132372), we conducted focus group meetings with patients receiving in-center hemodialysis and their family members to understand their knowledge and perceptions about: 1) CVD and arrhythmia in dialysis patients; 2) risk of death associated with CVD; 3) approaches to communicating the risk of CVD and arrhythmias; and 4) concerns about ICMs. We chose to conduct focus groups because little is known about patients’ knowledge and concerns about cardiac arrhythmia and how these might impact their acceptance of cardiac monitoring. We also sought to obtain feedback on the development of educational materials that would be used as part of a future research study in patients at risk of cardiac arrhythmia. We followed guidelines of the consolidated criteria for reporting qualitative research (CORE1) checklist in the design, execution, and analysis of focus groups.

*Participant Recruitment*

We recruited participants in-person from two community-based dialysis facilities in Baltimore, Maryland. Patients were eligible to participate if they were age 18 years or older, spoke English, and received in-center hemodialysis at one of the two dialysis facilities. None of the patients had any relationship to the study or research team members prior to study commencement. There was no restriction regarding time on in-center hemodialysis. Once the patients consented and enrolled, we then asked them to identify a family member or friend who was closely involved in their medical decisions to be approached for recruitment. All participants provided oral consent. The project was reviewed by the Johns Hopkins Medicine Institutional Review Board (IRB00133847) and designated as a quality improvement project which allowed waiver of written consent.

*Focus group conduct*

The focus group was guided by a moderator guide (see Supplemental Materials) which was developed by one of the study investigators (PLE). Two trained moderators who were study staff and not affiliated with the dialysis unit led the focus groups. Patient and family focus groups were held separately in a conference room at a local hospital and each session lasted approximately 90 minutes. After greetings and introductions, we asked participants open-ended questions designed to elicit their understanding of the risk of CVD and its complications in dialysis patients, as well as their thoughts on the use of ICMs. We audiotaped and transcribed verbatim all group discussions for data analysis.

*Analysis*

We used template analysis, a form of thematic qualitative analysis methods, to identify the core themes emerging from these discussions. Two reviewers independently reviewed the transcripts (LLB, CK) and grouped participant’s responses into themes and subthemes using a coding template developed from a subset of data that was refined during the coding process. After this initial review, both reviewers met to discuss their findings and developed consensus on the themes. In the event of discrepancy in adjudication, a third review (PLE) was identified a priori to adjudicate. These findings were then reviewed by other team members who provided critiques. We then selected emblematic quotations from the transcripts to illustrate each of the final themes.
